# Structure-guided development of an electrochemical aptasensor for *Salmonella* Typhi HlyE antigen detection using in silico and experimental approaches

**DOI:** 10.1038/s41598-026-38666-6

**Published:** 2026-02-26

**Authors:** Mohamad Ahmad Najib, Anja Winter, Khairul Mohd Fadzli Mustaffa, Muhammad Hafiznur Yunus, Eugene Boon Beng Ong, Kasturi Selvam, Muhammad Fazli Khalid, Mohd Syafiq Awang, Asrulnizam Abd Manaf, Yazmin Bustami, Habibah A. Wahab, Ismail Aziah

**Affiliations:** 1 Institute for Research in Molecular Medicine (INFORMM), Universiti Sains Malaysia, Kubang Kerian, 16150 Kelantan Malaysia; 2 School of Life Sciences, Keele University, Staffordshire, UK; 3 Institute for Research in Molecular Medicine (INFORMM), Universiti Sains Malaysia, Gelugor, 11800 Pulau Pinang Malaysia; 4 Collaborative Microelectronic Design Excellence Centre (CEDEC), Universiti Sains Malaysia, Bayan Lepas, 11900 Pulau Pinang Malaysia; 5 School of Biological Sciences, Universiti Sains Malaysia, Gelugor, 11800 Pulau Pinang Malaysia; 6 School of Pharmaceutical Sciences, Universiti Sains Malaysia, Gelugor, 11800 Pulau Pinang Malaysia

**Keywords:** Aptamer, Molecular docking, Molecular dynamics, Aptasensor, *Salmonella* Typhi, Biochemistry, Biological techniques, Biotechnology, Chemistry, Computational biology and bioinformatics, Drug discovery

## Abstract

Efficient and timely detection of biomarkers for typhoid fever is pivotal for improving treatment outcomes and preventing further transmission of the disease. This study addresses the pressing need for a rapid and accurate point-of-care test to detect *Salmonella* Typhi HlyE antigen, a vital biomarker for typhoid fever. Leveraging the growing prominence of computer-based simulations for aptamer-protein interactions modelling and electrochemical biosensors for their high sensitivity, this study presents the development of a novel electrochemical aptasensor designed to accurately and effectively detect *Salmonella* Typhi HlyE antigen in blood samples of typhoid patients. We previously identified and characterized selective aptamers against HlyE antigen, and identified AptHlyE97 as the aptamer with highest binding affinity and selectivity. In this study we showed that AptHlyE97 formed the most stable complex with HlyE in molecular docking and dynamics simulations and an unbound 5’ end to enable conjugation to the sensor surface. The aptasensor features AptHlyE97 conjugated via thiol immobilization to the surface of a screen-printed gold electrode, and utilizing potassium ferricyanide and potassium ferrocyanide for electrochemical detection. The aptasensor showed a significant (*p* < 0.001) square wave voltammetry (SWV) response to HlyE with a diagnostic performance of 100% sensitivity and 85.7% specificity against other bacterial communicable disease pathogens. Additionally, the aptasensor exhibited a strong linear response with a limit of detection (LoD) of 0.158 ng/mL. We present here a novel diagnostic aptasensor capable of efficiently and selectively detecting the HlyE antigen from *Salmonella* Typhi. This aptasensor provides a robust foundation for future research in typhoid diagnosis and also highlights the potential impact in advancing point-of-care detection strategies in community settings.

**Supplementary Information:**

The online version contains supplementary material available at 10.1038/s41598-026-38666-6.

## Introduction

Typhoid fever, an invasive bacterial disease caused by the bacterium *Salmonella enterica* serotype Typhi remains a significant global health concern^1^. The disease poses serious health risks, particularly in developing countries of Africa and Asia, where access to safe water, improved sanitation and hygiene (WASH) infrastructure is limited^2^. Current gold standard for typhoid diagnosis, which involves the isolation and biochemical identification of bacterial cultures from blood and stool samples in laboratories are limited in terms of their speed and practicality for point-of-care settings^3^. In addition, this method also requires extensive sample preparation, skilled personnel and sophisticated laboratory equipment, which are not always available in resource-limited settings. Numerous studies in recent years underscore the urgent need for innovative diagnostic solutions that can provide rapid and accurate results^4–6^. These include serological tests like Typhidot and TUBEX, which detect antibodies against *S.* Typhi^7^. However, serological tests are often inaccurate due to cross-reactivity with other infections^8^. The result of the meta-analysis found TUBEX to have an average sensitivity of 78% and a specificity of 87%; Typhidot had an average sensitivity of 84% and a specificity of 79%, and Test-It (KIT) was found to have an average sensitivity of 69% and a specificity of 90%^9^. This highlights the need for alternative rapid detection methods with improved accuracy, sensitivity and specificity. One of such alternative to antibodies for diagnostic applications is provided by utilizing aptamers to specifically bind to target antigens.

Aptamers are short, single-stranded DNA or RNA molecules that can selectively bind to specific targets and exhibit high affinity and specificity for their targets, thus making them suitable for use in diagnostic assays^10^. The advantages of aptamers include their high thermal stability, ease of synthesis on a large scale with low cost, while retaining high reproducibility and reliability^11^. Recent research has demonstrated the application of aptamers in various diagnostic platforms, including lateral flow assays^12^, enzyme-linked immunosorbent assays^13^ and electrochemical biosensors^14^. We have recently identified specific aptamers against the HlyE antigen of *S.* Typhi, with AptHlyE97 demonstrating a Kd value of 83.6 nM and high specificity against other bacterial species such as *Salmonella* Paratyphi A, *Salmonella* Paratyphi B, *Shigella flexneri*, *Klebsiella pneumoniae* and *Escherichia coli*^15^. However, understanding the aptamers’ binding interaction with their target antigen is crucial prior to utilization in diagnostic devices. Therefore, in silico computational approaches such as molecular docking and molecular dynamic simulations play an important role in providing information on the specific binding interaction of the aptamer to the target antigen, the key interactions stabilizing the aptamer-antigen complex and the conformational changes in the aptamer upon binding. This information enables us to identify whether immobilization of the aptamer on an electrode surface would retain its function^16^, as well as guide the selection of the optimal immobilization site on the aptamer to preserve its binding activity and help design aptamer variants with enhanced affinity and specificity through strategic mutations^17^.

Electrochemical aptasensors are a fundamental tool of biosensing technology able to leverage the superior characterstics of aptamers^18^. They detect target-induced conformational changes in aptamers, which alters the electrochemical signal in response to the binding of specific molecules. This detection method is typically performed in an electrochemical cell, utilizing various electrode configurations such as two-electrode and three-electrode systems to facilitate the measurement of electrical signals generated during the interaction between the aptamer and its target^19^. Unsurprisingly, electrochemical aptasensors have recently gained attention due to their ability to provide rapid, sensitive and label-free detection of target analytes^12^. Therefore, the development of an electrochemical point-of-care testing (POCT) aptasensor targeting the *S.* Typhi HlyE antigen, a vital biomarker for typhoid fever, is urgently required and presents a promising alternative to currently available devices with improved sensitivity, speed, ease of transport and storage, low cost and powerful functionality.

**Fig. 1:**
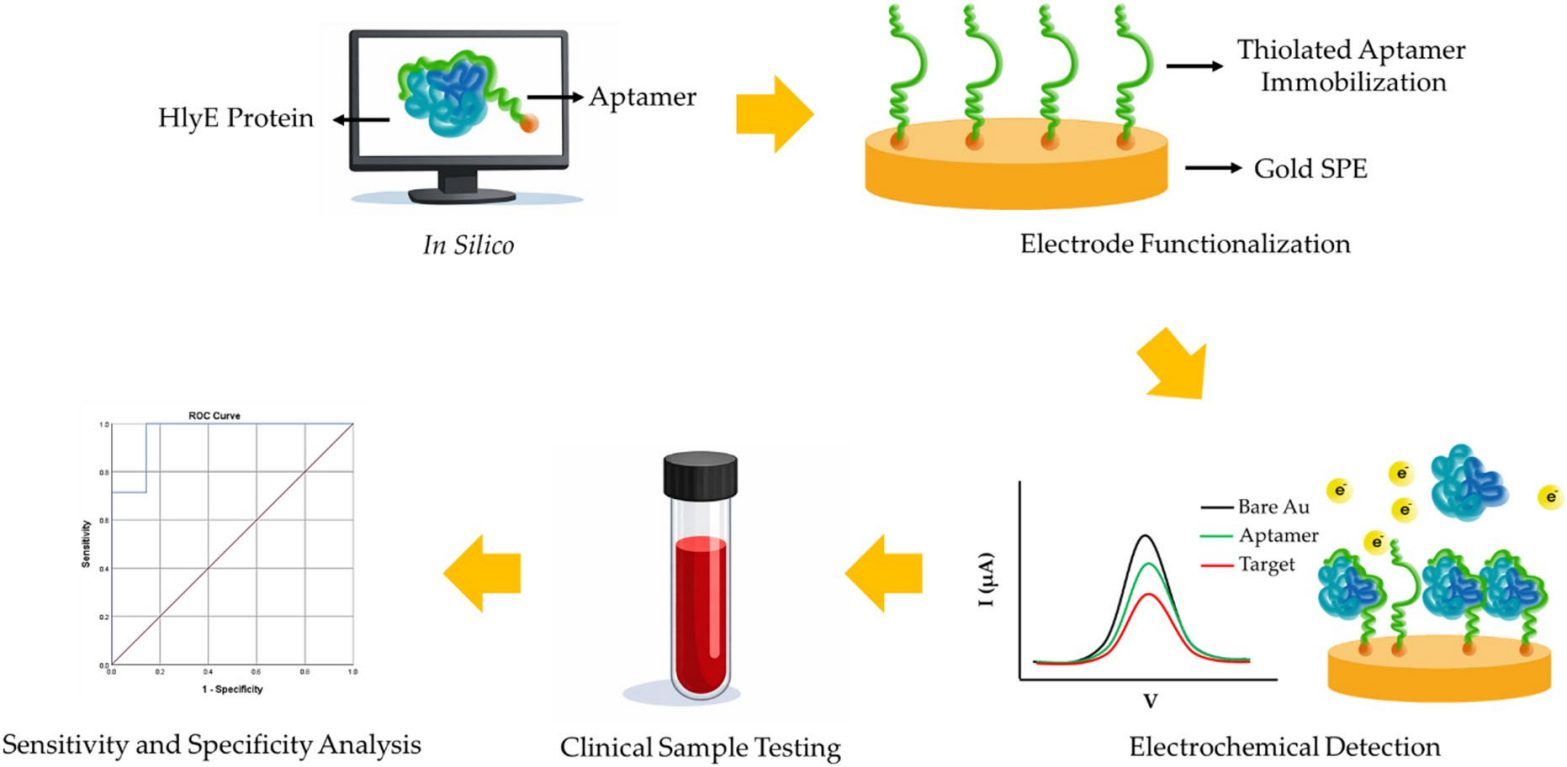
Schematic diagram illustrating the overall workflow of the study.

Here we address these challenges by creating a reliable and efficient diagnostic tool that can significantly improve the management and control of typhoid fever outbreaks in community settings. A schematic diagram illustrating the overall workflow of the study is shown in Fig. 1. We utilized our previously identified nanomolar affinity aptamer AptHlyE97 (Table S1) as the molecular recognition element and conjugated it with a thiolated group at its 5’-end to enable the immobilization on the gold working electrode (WE) surface^20^. The electrochemical responses examined using square wave voltammetry (SWV) confirmed successful aptasensor fabrication using a 1 µM immobilized aptamer and demonstrated the ability of the sensor to detect the HlyE antigen at concentrations as low as 0.5 µg/mL. Furthermore, our aptasensor was effective in distinguishing between different bacterial lysates, *S*. Typhi, *S*. Paratyphi A, *S*. Paratyphi B, *E. coli*,* K. pneumoniae* and *S. flexneri*, with a ~10% lower current reduction. Finally, the feasibility of the newly developed electrochemical aptasensor was evaluated using serum samples of typhoid patients, healthy individual and other bacterial diseases patients, demonstrating a sensitivity of 100% and a specificity of 85.7%. ROC analysis determining AUC with 0.959 suggesting that the aptasensor has a high ability to correctly identify typhoid patients compared to healthy individuals and those with other diseases, and is therefore indicates very good diagnostic performance^21^.

## Materials and methods

### Protein production and aptamer

HlyE was expressed in BL21 (DE3) purchased from New England Biolabs, USA, using a construct of hlyE gene clones into pET28a as reported in Ref. 22 and purified using IMAC as reported in previous study^15^. Size exclusion chromatography was carried out using PBS with 1% glycerol on a Superdex 75 column purchased from Cytiva, Sweden (Figure S1). The Aptamer Apt_HlyE97 was synthesized using MWG Eurofins and used as delivered.

## Bacterial lysate Preparation

Bacterial strains of *Salmonella* Typhi, *S*. Paratyphi A, *S*. Paratyphi B, *Escherichia* coli, *Klebsiella* pneumoniae, and *Shigella* flexneri were obtained from cultures prepared in a previous study^15^. These bacterial cells were subjected to lysis by incubating the suspension at at 95 °C for 5 min. After heat treatment, the lysate was centrifuged at at 10,000 × g for 5 min to pellet cellular debris. The supernatants containing the bacterial lysates were collected, and the total protein concentration of each lysate was quantified using a NanoDrop spectrophotometer. Prior to electrochemical measurements, all lysates were normalized to the same total protein concentration to ensure that differences in current responses were attributable to specific aptamer–HlyE interactions rather than variations in protein content. The normalized lysates were stored at −20 °C until further use.

### Ethical statement

This study utilized archived human serum samples previously collected and stored for research purposes. Ethical approval for the use of these serum samples was obtained from the Human Research Ethics Committee of Universiti Sains Malaysia (JEPeM), under the approval code USM/JEPeM/KK/25,010,104. All experimental procedures were performed in accordance with the relevant guidelines and regulations. Informed consent was obtained from all subjects or their legal guardians prior to sample collection and participation in the study.

### Small angle X-ray scattering (SAXS)

SAXS was performed at the B21 beamline (Diamond Light Source Ltd., Didcot, United Kingdom, MX-34438-187). B21 was equipped with Eiger 4 M detector (DECTRIS Ltd., Baden-Daettwil, Switzerland), the λ of the beam was 1Å with q ranging from 0.0045 to 0.45 Å^−1^. To account for concentration effects, samples were subjected to a 1:2 dilution series with initial concentrations of 10 mg/ml for Apt_Hly97, 11.56 mg/ml for HlyE protein and 5.5 mg/ml of HlyE: Apt_Hly97 complex (assumed stoichiometry 1:1).

SAXS data was collected as 10 × 1s exposure on 35 µl samples at 25 °C. A continuous flow cell capillary was used to reduce radiation damage. The data was averaged, the frames compared, and those displaying significant alterations discarded. SAXS profiles were subtracted by the corresponding blanks, and ATSAS^23^ used to estimate the radius of gyration (R_g_) in the Guinier Region where qR_g_ < 1.3, to calculate the pair distance distribution functions *P*(*r*) as well as the molecular weight using the Guinier analysis from *I*(0).

### *Ab initio* shape determination

SAXS data were fitted using GNOM^24^, and *ab initio* shape modelling conducted using DAMMIF^25^. For DAMMIF, 20 iterations of the algorithm were performed with no symmetry or anisometry. The *P*(*r*) data were used as an input for fast DAMMIF modeling to create 10 initial dummy atom models. Alignment, clustering, selection, averaging and filtering of the ten runs were performed using the automatic algorithm provided in the ATSAS Primus pipeline^23^. The HlyE model, Apt_HlyE97 model and model of the complex were used to calculate the theoretical scattering profiles using (PEPSI) modelling server^26^.

### Molecular docking

Molecular docking was performed using AutoDock Vina to investigate the interaction between the DNA aptamers and the *S.* Typhi HlyE antigen^27,28^. The aptamers were prepared as ligand. Initially, water molecules were removed and polar hydrogen atoms were added and its nonpolar hydrogen atoms were merged using AutoDock Tools^29^. Then, Kollman Charges were added and Gasteiger Charges were computed. For the aptamers, all bonds were set as rotatable. For macromolecule preparation, *S.* Typhi HlyE antigen was selected as the macromolecule. Subsequently, a grid box with a dimension of 90 × 50 × 100 was defined around the *S.* Typhi HlyE antigen to encompass potential binding interactions with the ligand. The configuration file was prepared with input parameters including the macromolecule and ligand structures, grid box dimensions, centre of x, y, z dimensions and search space parameters such as number of modes^10^, energy range^3^ and exhaustiveness^8^ and molecular docking was performed using AutoDock Vina. PyMOL was utilized for docking result visualization.

### Molecular dynamics (MD) simulation of complexes

Molecular dynamics (MD) simulation was carried out using the complex obtained from docking using AutoDock Vina^27,28^. The system was prepared using CHARMM-GUI solution builder for system solvation, ionization, and neutralization^30,31^. CHARMM36 force field were applied for system parametrization. The molecular dynamics simulations were performed with an integration step of 2 fs and coordinates of the simulation model were recorded per 1 ps employing GROMACS software (version 2.1) to elucidate the dynamic behavior of aptamer-antigen complex^32^. The starting structures were immersed in a dodecahedron box of simple point charge (SPC) water model and neutralized with sodium and chloride ions. The box dimensions were chosen to provide at least 10 Å buffers of solvent molecules around the solute. Subsequently, the system was relaxed by energy minimization for 1 ns followed by 500 ps equilibration. MD simulation was carried out for 100 ns at 298.15 K temperature and 1 bar pressure. The resulting trajectories were analyzed for the root mean square deviation (RMSD) values.

### Preparation of screen-printed gold electrodes (Au-SPE)

The Au-SPE (DRP-220AT SPE) used in this study were purchased from Metrohm DropSens (Oviedo, Spain). The Au-SPE consisted of a gold working electrode (WE) of 4 mm in diameter, a gold counter electrode (CE) and a silver reference electrode (RE). All measurements were performed using a potentiostat (µStat-i 400s, Metrohm DropSens, Oviedo, Spain) at room temperature (RT) (25 °C) controlled by Dropview software version 2.0. The Au-SPE was electrochemically activated by cyclic voltammetry (CV) scanning with a potential range from 0 V to 1.6 V in 0.5 M sulfuric acid (H_2_SO_4_) at a scan rate of 100 mV/s and Estep of 2 mV for 15 cycles prior to use^33^.

### Immobilization of the aptamer onto Au-SPE

Before immobilizing the aptamer on the electrode, 100 µL of 2 µM thiol-modified aptamer (5’-(CH_2_)_6_-SH-AptHlyE97) was reduced by incubating in 100 µL of 100 µM tris-(2-carboxyethyl) phosphine hydrochloride (TCEP) for 2 h at RT in dark to reduce the disulfide bonds (S-S) to free thiol groups (–SH) by cleaving the disulfide linkage^34^. The reduced aptamer was denatured at 95 °C for 5 min and cooled at RT for 30 min to obtain its stable conformation. Subsequently, 5 µL of the reduced aptamer was immobilized on the WE surface overnight (16 h) at RT. Afterward, the electrode was washed with deionized water to remove the unbound aptamers and then backfilled with 5 µL of 2 mM 6-mercapto-1-hexanol (MCH) for 2 h at RT^34^. Finally, the electrode was rinsed with deionized water to remove the unbound MCH.

### Electrochemical detection

Square wave voltammetry (SWV) was employed as the electrochemical detection method. The SWV measurements were performed with a scanning potential range from − 0.15 to 0.5 V at a frequency of 20 Hz, step size of 5 mV and an amplitude of 2 mV. All measurements were performed at RT in 100 µL of 5 mM potassium ferricyanide K_3_(Fe(CN)_6_ and potassium ferrocyanide K_4_(Fe(CN)_6_ diluted in PBS (0.1 M, pH 7.4). The decrease of the peak currents (∆I) was defined as the different currents in the absence and presence of the target antigen measured by SWV and was calculated by:

$$\:\varDelta\:I={I}_{0}-I$$

∆I: Decrease of peak current.

I_0_: Current measured in the absence of the target antigen by SWV.

I: Current measured in the presence of the target antigen by SWV.

The signal loss is the percentage of decrease of peak current compared to the current measured in the absence and presence of the target bacterium and was calculated as:

$$\:Signalloss=\frac{\varDelta\:I}{{I}_{0}}\:\times\:100$$

### Statistical analysis

All experiments and measurements were conducted in triplicate. Data were analyzed using GraphPad Prism version 9. The data are expressed as the mean ± standard deviation (SD) when appropriate with a p-value of less than 0.05 is considered as statistically significant difference. Receiver Operating Characteristic (ROC) analysis was performed using SPSS version 26.0 (IBM Corporation) to evaluate the diagnostic performance of the developed electrochemical aptasensor in distinguishing *S*. Typhi-positive and negative serum samples. ROC analysis is a graphical method that assesses the trade-off between sensitivity and specificity across a range of decision thresholds, providing a comprehensive evaluation of classifier performance. The area under the ROC curve (AUC) was calculated as a quantitative measure of overall diagnostic accuracy, where an AUC value of 1.0 indicates perfect discrimination and an AUC of 0.5 indicates no discriminative ability. Higher AUC values reflect better sensor performance in differentiating between positive and negative samples. The optimal cut-off value was determined based on the ROC curve by maximizing the Youden index (Sensitivity + Specificity − 1), which identifies the threshold that provides the best balance between sensitivity and specificity. Based on this analysis, a current response of 82.8 µA was selected as the optimal cut-off value for classifying samples as *S*. Typhi positive or negative. Using blood culture as the reference standard, the sensitivity and specificity of the developed electrochemical aptasensor were calculated using equation as follows:

$$\:Sensitivity=\frac{True\:positives\:\left(TP\right)}{True\:positives\:\left(TP\right)+False\:negatives\:\left(FN\right)}$$

$$\:Specificity=\frac{True\:negatives\:\left(TN\right)}{True\:negatives\:\left(TN\right)+False\:positives\:\left(FP\right)}$$

The limit of detection (LoD) and the limit of quantification (LoQ) of the aptasensor were calculated based on the IUPAC guidelines^35^. The equation for the LoD and LoQ calculation are as follows:

$$\:LoD=\:3\times\:\frac{Standard\:deviation\:of\:the\:blank}{Slope\:of\:the\:regression\:line\:}$$

$$\:LoQ=\:3\times\:LoD$$

## Results and discussion

### *In silico* analysis of the interaction between aptamers and Hlye

Our previously generated structure prediction of *S.* Typhi HlyE antigen tertiary structure using AlphaFold^36^ revealed an all-helical protein apart from two short antiparallel β-strands which is in agreement with other HlyE structures that have been crystalized from *E.coli* (PDB: 1QOY, 4PHQ, 6MRT)^15^. The modelled HlyE protein structure was energy optimised using GROMACS^32^, to remove any steric clashes and refine the geometry of the structure. This step ensures that the protein conformation resides in a local energy minimum, thereby increasing the stability and reliability of the predicted model for subsequent molecular docking and dynamics studies.

**Fig. 2:**
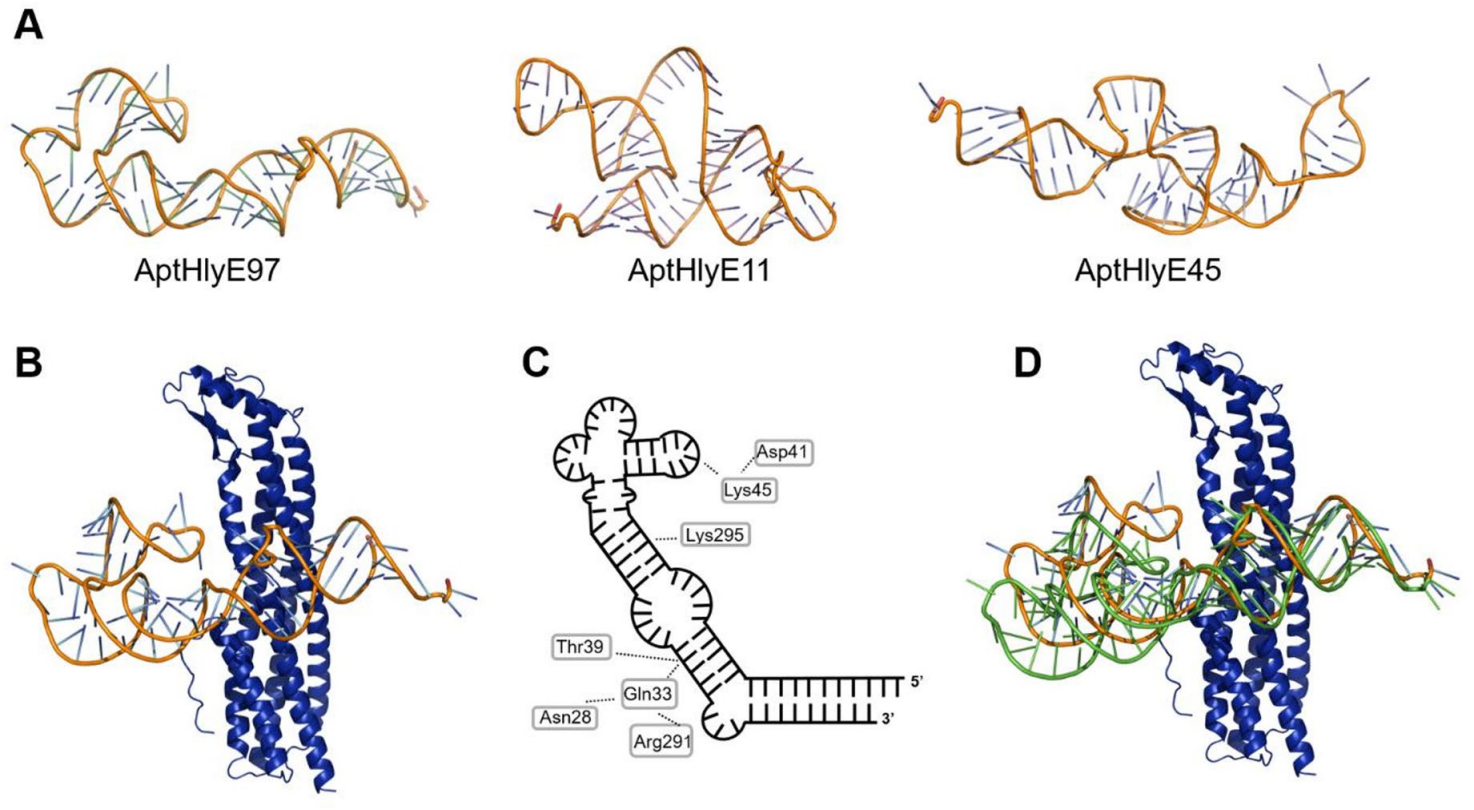
Molecular modelling of HlyE aptamers and protein: aptamer complex. (**A**) 3D models of AptHlyE97, AptHlyE45 and AptHlyE11. (**B**) Model of AptHlyE97-HlyE complex showing a ‘wrap-around’ of the AptHlyE97 (orange, coloured by element) and S. Typhi HlyE antigen (blue). (**C**) Comparison between bound (orange) and unbound (green) Apt97 revealing a movement of 11Å (angle of ~14°) upon binding to HlyE (blue). (**D**) Interaction map of Apt97 with HlyE residues.

Tertiary structures of our previously identified aptamers AptHlyE11, AptHlyE45 and AptHlyE97 were prepared using RNAComposer^37^ and energy optimised using GROMACS^32^. The energy-optimized predicted 3D structures of the aptamers are diverse, with Apt97 and Apt45 being more elongated and showing several intra-molecular interactions, whereas Apt11 has a more globular structure with large flexible single-stranded loops (Fig. 2A). AptHlyE97 was identified as the best binder in our previous study^15^, therefore we examined its predicted 3D structure more closely. Several intra-molecular hydrogen bonds between bases that form the stalk are apparent: A83:G1, C82:G2, G81:C3, C80:G4, T79:A5, T78:A6, A77:T7, A76:T8, G75:C9, followed by a loop of TGG, where N7 of G12 interacts with an oxygen on the backbone phosphate of A68. This is followed by another stretch of base pairing between C74:G13; C73:G14, G72:C15, A71:G16, T70:A17, followed by an unordered region where no true base pairing occurs, between bases 69−65 and 18–22. This unordered region allows a distortion and slight bending of the otherwise quite well-paired first part of the aptamer. This is followed by a last stretch of paired bases: G64:C23, G63:C24, A62:t25, G61:A26, G60:G28. G27 is flipped out of plane and interacts with G30 and C58. The remaining bases of the aptamer form little true base pairs and are mainly organised in three flexible loops, apart from G59:C29 and C57:G31, but rather a varied set of hydrogen bond interactions with both base atom as well as backbone oxygens. The large loop between G32 and G44 was predicted to force some bases to flip out of plane (e.g. T35, T40, T41, C51, C52), with a hydrogen bond between G32 andA37 causing a further ‘kink’ in the aptamer. The remaining bases of the loop form several interactions, but with only three potential base pairings between A45:T56, C46:G55, C47:G54. The interactions of N3 of G47 with O5 of T25 and N2 of G55 with OP1 on G55, stabilised by a hydrogen network between G48, G49, G51 and G53, bend the loop against the latter part of the aptamer thus stabilising the ‘kink’.

Molecular docking of the antigen HlyE and aptamers using AutoDock Vina and subsequent molecular dynamics simulation and energy analysis using GROMACS^32^ showed negative calculated binding energies for all three aptamers-HlyE complexes, −9.8 kcal/mol for AptHlyE11, −13.9 kcal/mol for AptHlyE45 and −15.5 kcal/mol for AptHlyE97, respectively. This indicates a high stability of the interaction between the aptamers and the target antigen, particularly for AptHlyE97 (Table 1), which indicates that AptHlyE97 likely engages in highly favorable interactions with the HlyE antigen. This is consistent with our previous findings from the Enzyme-Linked Oligonucleotide Assay (ELONA), where AptHlyE97 exhibited the highest dissociation constant (Kd) value of 83.6 nM^15^. This agreement between the molecular docking results and ELONA assay outcomes reinforces the results of our docking predictions and highlights AptHlyE97’s superior binding characteristics. This excellent correlation was also found in other studies^38^ and further validates our approach.

Examination of the predicted complex between HlyE and AptHlyE97 revealed that the aptamer “wraps around” the 5-helix bundle of HlyE (Fig. 1B), limiting the interaction between the aptamer AptHlyE97 (blue) and *S*. Typhi HlyE antigen (yellow) to only certain parts of each molecule. Main interactions are formed between Lys45 and OP1 and OP2 of G50 where the side chain position of Lys45 is stabilised by interaction with OD2 of Asp41 (Fig. 2C). Three interactions are formed between G16 and the protein: OP1 of G16 with HG1of Thr39, H3 of G16 with the main chain oxygen of Gln 33 and H21 of G16 with OE1 of Gln 33. The interaction between Gln33 and G16 is further supported by the interaction of Gln33 1HE2 interacting with the main chain oxygen of Asn28 and NH1 of Arg291. Lys295 is shown as fully protonated in structure but could interacting with H3 of A62 in its deprotonated form.

Comparing the bound and unbound aptamer structures, it becomes apparent that the ‘kink’ in the aptamer grants the required flexibility to facilitate binding to HlyE, with a movement of 11Å or 14.5° observed (Fig. 2D). One might speculate that the high binding affinity between AptHlyE97 and HlyE is facilitated by the aptamer adopting a conformation in its unbound state which is close to the bound conformation therefore reducing the energy needed for binding. Notably, the 5’ end and 3’ end of AptHlyE97 (stalk) does not interact with the target antigen, highlighting its suitability for conjugation with thiol groups, which is prerequisite for immobilization onto the sensor surface.

**Table 1:** Comparison of AutoDock Vina Docking scores vs. Kd of the ssDNA aptamers against HlyE obtained through ELONA.

Aptamers-HlyE complex-	Free energy of binding	Kd (ELONA)
AptHlyE11	− 9.8 Kcal/mol	102.2 nM
AptHlyE45	− 13.9 Kcal/mol	119.3 nM
AptHlyE97	− 15.5 Kcal/mol	83.6 nM

The stability of the systems was evaluated by assessing the all-atom RMSD values of the three simulated systems [HlyE: AptHlyE11 (complex11), HlyE: AptHlyE45 (complex45), and HlyE: AptHlyE97 (complex97)] during 100 ns MD simulations (Figure S2) using GROMACS^32^. The RMSD of complex11 reached an equilibrium with a stable RMSD value of ~2.0 Å after 10 ns with similar fluctuations throughout the 100 ns (Fig. 2B, green). Meanwhile, for complex45, the RMSD values were fluctuated in the range of ~2.0 Å to 3.0 Å throughout the 100 ns (Fig. 2B, red). The RMSD of complex97 reached an equilibrium with a stable RMSD value of ~1.5 Å to 2.5 Å after 45 ns and then increased slightly to 3.0 Å with similar fluctuations throughout the 100 ns (Fig. 2B, black).

### SAXS validation of the obtained models

**Fig. 3:**
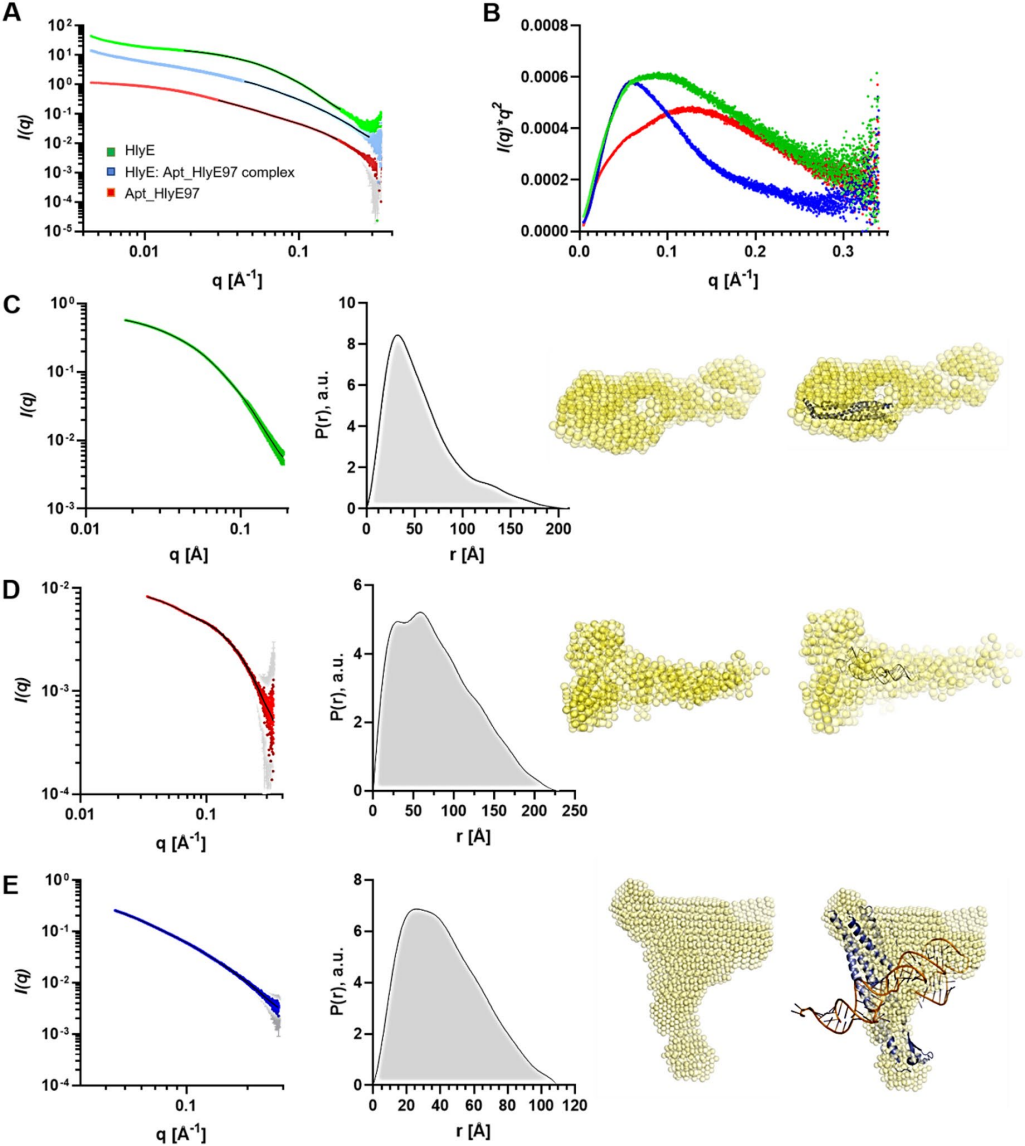
SAXS analysis of HlyE protein, Apt_HlyE97 and their complex. (**A**) SAXS profiles of HlyE protein (green), Apt_HlyE97 (red) and their complex (blue) with data fit obtained with GNOM^40^ (black lines). (**B**) Kratky plots of SAXS data for HlyE protein (green), Apt_HlyE97 (red) and their complex (blue). (**C**) SAXS profile of HlyE with GNOM fit (left), P(r) as distribution of the intraparticle distances between scattering centers (middle), and converged weighted bead model obtained using DAMMIF^25^. (**D**) SAXS profile of Apt_HlyE97 with GNOM fit (left), P(r) as distribution of the intraparticle distances between scattering centers (middle), 10 bead models obtained using DAMMIF overlaid. (**E**) SAXS profile of the complex between HlyE amd Apt_HlyE97 with GNOM fit (left), P(r) as distribution of the intraparticle distances between scattering centers (middle), and converged weighted bead model obtained using DAMMIF.

SAXS was used to ascertain the tertiary, spatial structure and thus characterize the physical parameters of HlyE, AptHlyE97 and their complex, determine its dispersed state such as monodispersity, as well as size and molecular weight of the particles in solution. All samples produced consistent scattering profiles over several protein concentrations, however, from three concentrations submitted to SAXS we use the intermediate concentration at 5 mg/ml as it showed less aggregation at low-*q* values compared to 10 mg/ml while providing better signal-to-noise ratios than 2.5 mg/ml. Generally, the scattering signal in the region of 0.01 or 0.03 < *q* < 0.34 Å^−1^ was used for further analysis to avoid the low-*q* region. HlyE and the HlyE: aptamer complex in particular show signs of aggregation with a *q*^− 1^ upward slope in intensity present in the low-*q* region < 0.01Å^−1^ (Fig. 3A). This was also confirmed in the Kratky plot which showed an upturn at low-*q* values (Fig. 3B). Qualitative analysis of the three SAXS profiles shows they are distinct with a visible bump present for the HlyE: aptamer complex around 0.05Å^−1^ and a flattened curve for the aptamer in that region. The Kratky plots also show that all three particles are well formed and globular in shape, as evidenced by the appearance of bell-shaped curves, but also contain flexible elements to their structure because the curve does not return to zero at high-*q* values. The HlyE: aptamer complex is more compact and smaller than the HlyE protein, as its bell-shaped curve is less broad and has a peak at lower *q* (Fig. 3B, blue and green curves) compared to the complex sample. This is at first surprising but could indicate oligomer formation of the protein. Indeed, gel filtration suggests that HlyE protein appears as oligomers, likely dimers in solution, confirming findings from SAXS (Figure S3). The Kratky plot of Apt_HlyE97 shows a broad bell-shaped curve indicating a folded and somewhat compact structure of the molecule but one could also deduce that the broadness or possibly shoulder in this plot may indicate a more complex shape reflecting the contributions of different structural elements. Therefore, we expect the aptamer to present with a quite loose 3D structure that is quite flexible. This may impact modelling and needs to be kept in mind for further analysis.

The linear part of the low-*q* region (*q*Rg < 1.3) was subjected to Guinier analysis and revealed Rg values for HlyE and Apt_HlyE97 at 48Å and 64 Å, respectively, and a smaller Rg for the complex of 33.6Å. The large Rg of the aptamer compared to the protein and complex, despite having a smaller molecular weight, indicates an elongated and likely highly flexible conformation of the aptamer. Our data suggests that the aptamer does not form a complex shape as was observed from other aptamers in SAXS^39^, and is likely gaining structural complexity upon binding to HlyE. The comparatively small Rg for the protein-aptamer complex confirms first observations from the Kratky plot, and may point to a conformational change in HlyE upon binding to its aptamer. The indirect Fourier transform of the SAXS curve to determine P(r) revealed that HlyE has a maximum distance Dmax of 194Å with a most likely intraparticle distance between scattering centers at 32Å, suggesting an elongated particle (Fig. 3C). P(r) for Apt_HlyE97 shows two peaks, one at 29Å which is slightly larger than the width of double-stranded DNA, and a second one at 59Å, with a Dmax at 230Å (Fig. 3D). Seeing as two peaks are present, it seems unlikely that the DNA aptamer is simply folded in half to allow base pairing, but would rather possess a complex 3D structure with double-helix and a protrusion. This loose structure may not be well defined, indicating a flexible aptamer with only partial base pairing. The SAXS profile of the complex between HlyE and aptamer resulted in a good fit in GNOM and returned P(r) with a broad peak at around 24Å to 37Å and a Dmax at 109Å which may indicate a complex, possibly flattened structure rather than globular (Fig. 3E).

Next, 3-dimensional bead models of the overall electron density of each particle was reconstructed in the program DAMMIF^25^. 10 models were calculated, averaged and weighted to produce a final model for each sample. HlyE produced a good model with a final χ^2^ value of 0.1097. However, it is evident that the density is too large for just one HlyE molecules and, taken together with a slightly larger apparent molecular weight in size exclusion chromatography, indicates oligomer formation in solution, either side-to-side or end-to-end. This is not unusual for all-helical proteins in solution, and in the case of HlyE could be part of its function as a pore-forming toxin. The already indicated disorder in the aptamer was confirmed in the bead modelling, where the modelling process did not converge on any distinct conformation. In addition, the modelled density is larger than the Apt_HlyE97 model and indicates a highly flexible structure. Nonetheless, two protrusions can be seen, corresponding to the different intra-particle distances observed. The HlyE: Apt_HlyE97 complex presents with a quite compact, flat structure and a reasonable solution for bead modelling with a probability that the model fits the data of 0.595. The model also confirms that the complex consists of one molecule HlyE bound with one aptamer. Therefore, it is likely that the protein HlyE and the aptamer undergo conformational changes upon complex formation where the protein returns to a supported monomeric state and the aptamer adopts a secondary, and likely tertiary, structure. The bead modelling of our SAXS data furthermore show that the *in silico* modelling of the complex was successful and likely represents the complex structure.

For comparison, the PEPSI server^26^ was applied to the models of HlyE, HlyE in complex with Apt_HlyE97 and Apt_HlyE97 alone in order to independently validate them against the SAXS data. Comparison of theoretical with experimental curves generally fit well, yet displayed some differences in the mid-to high-*Q* range around 0.1Å^−1^, which could be due to some flexibility in 3D structure and differences between the atomic model and experimental data. The largest difference can be observed between the theoretical and experimental profiles of HlyE protein. This is most likely due to oligomer formation in HlyE, which was not taken into account when generating the theoretical SAXS profile from a modelled HlyE monomer, but confirms on the other hand that HlyE indeed forms oligomers in solution. Differences are also observed between theoretical SAXS profiles, where HlyE shows a bump around 0.1 Å^−1^ whereas the SAXS profile for Apt_HlyE97 is rather flat in this region and the theoretical profile for the complex shows a dip. This shows that all three species would produce distinct SAXS profile, which is reflected in our experimental data. The χ^2^ values between experimental data and theoretical SAXS profiles are summarized in Supplementary Table S2^26^.

### Development of the aptasensor

**Fig. 4:**
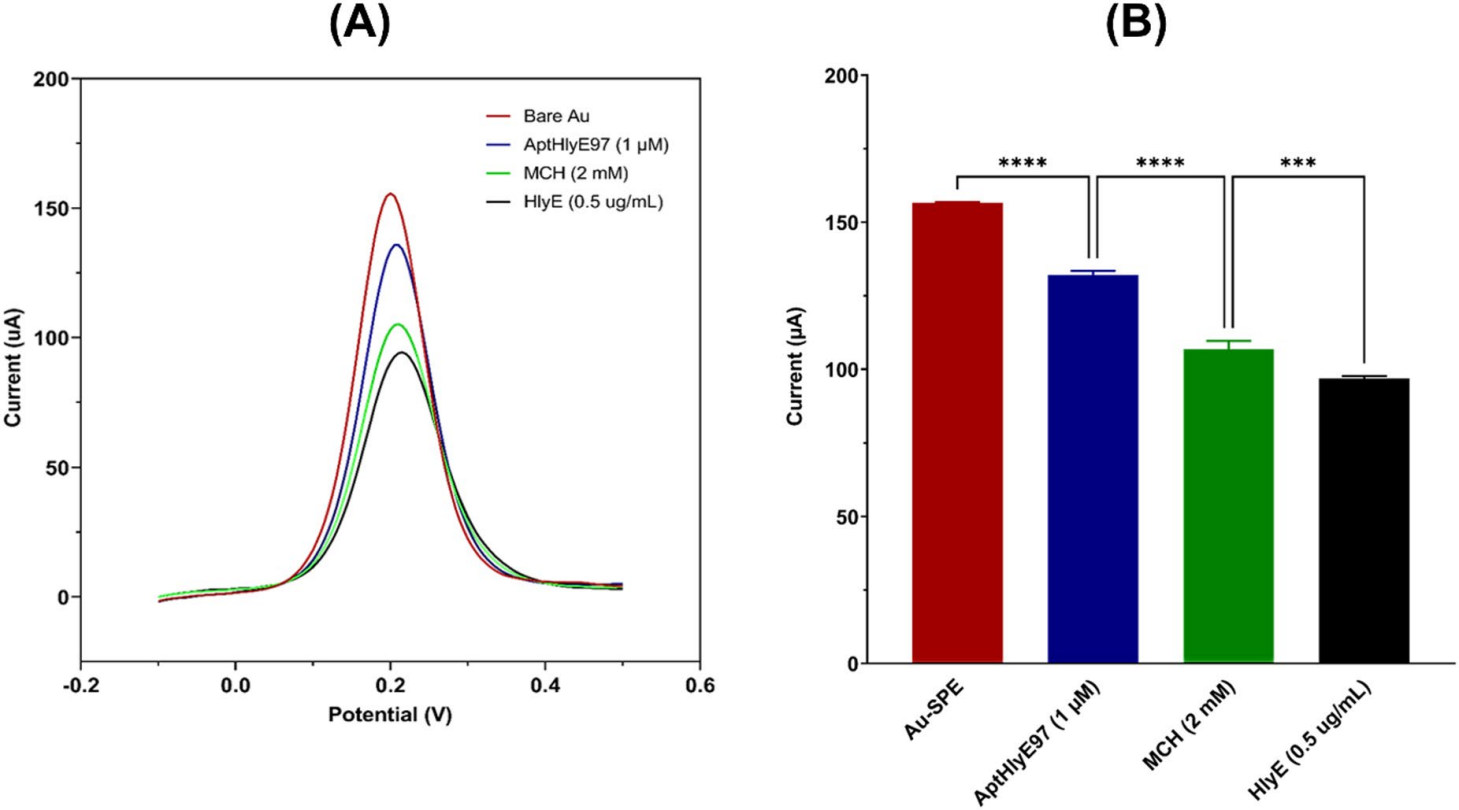
(**A**) Square wave voltammetry responses for the bare Au-SPE (red), after immobilization with 1 µM aptamer (blue), after blocking with 2 mM MCH (green) and after adding 5 µL of 0.5 µg/mL recombinant *S.* Typhi HlyE antigen (black). Each step demonstrates the functionalization and subsequent antigen detection capabilities of the aptasensor. (**B**) Bar graph illustrating the peak SWV responses (µA) of the aptasensor. Each data point represents the averaged results of three replicates ± the standard deviation. Statistical analysis was conducted using the One-way ANOVA and post-hoc test (Tukey’s Test). **** denotes *p* < 0.001 and *** denotes *p* < 0.01.

To the best of our knowledge, this study presents the first electrochemical aptasensor utilizing aptamer to detect HlyE antigen as biomarkers associated with typhoid fever in labeled-free systems. Previous research has predominantly concentrated on other serotypes of *Salmonella*, with most studies developing aptasensors aimed at food screening rather than clinical applications^41^. Initially, the Au-SPEs were fabricated and evaluated using SWV to investigate their electrochemical behavior at various stages of functionalization and target incubation (Fig. 4A). The bare Au-SPE exhibited SWV peak current of 156.6 µA, serving as the baseline response and reflecting efficient electron transfer between the redox probe and the electrode surface. Following the baseline measurement, the Au-SPE were modified by immobilizing a 1 µM of aptamer. For the development of the aptasensor, AptHlyE97 was selected due to its demonstrated highest binding affinity for the *S*. Typhi HlyE antigen, as evidenced by ELONA and molecular docking studies. The peak current of SWV response after aptamer immobilization was found to be 132.1 µA. This represents a current reduction of 24.5 µA, equivalent to a 16% of signal loss, indicating successful aptamer immobilization on the electrode surface (Fig. 4B). This decrease is attributed to the formation of a negatively charged aptamer layer on the electrode surface, which partially impedes electron transfer by introducing steric hindrance and electrostatic repulsion to the redox species. Subsequently, the Au-SPE were blocked using a 2 mM of MCH to prevent non-specific binding. The peak current of SWV response after MCH blocking was 106.8 µA. This step resulted in an additional current reduction of 25.3 µA, corresponding to a 19% of signal loss from the previous step (Fig. 4B). This further signal attenuation is consistent with the formation of a compact and well-organized self-assembled monolayer, which increases surface coverage and further restricts electron transfer, indicating effective passivation of the electrode surface^42^. Finally, the Au-SPE were incubated with the target analyte at a concentration of 0.5 µg/mL. The SWV response following target incubation was 96.8 µA. This corresponds to a current reduction of 10 µA from the MCH-blocked state, which equates to a 10% of signal loss (Fig. 4B). This final decrease is attributed to the formation of an insulating aptamer–antigen complex on the electrode surface, which further hinders electron transfer and confirms specific binding of the HlyE antigen to the immobilized aptamer.

### Optimization of the aptamer concentration

**Fig. 5:**
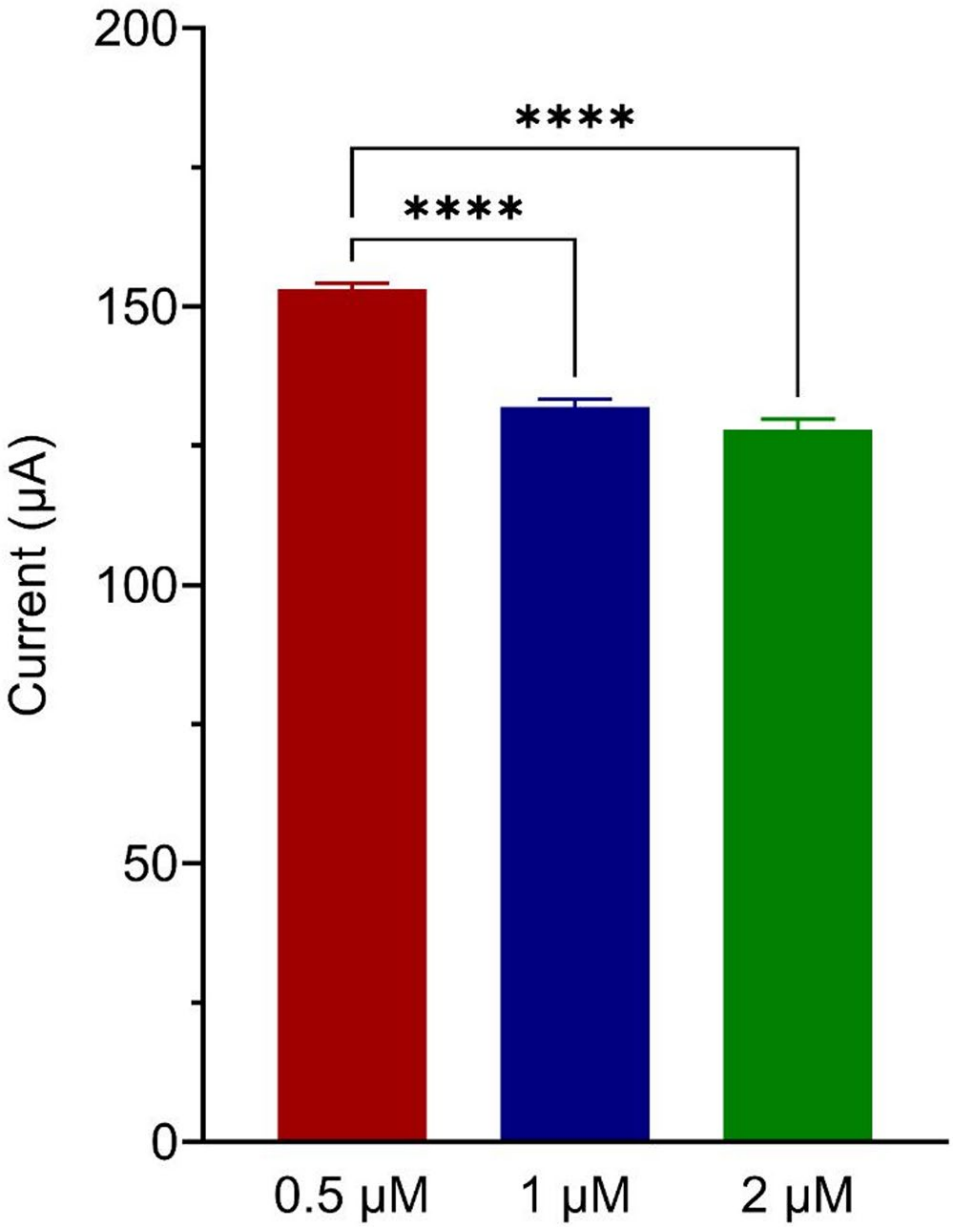
Bar graph illustrating the square wave voltammetry (SWV) response on different concentrations of aptamer. Each data point represents the averaged results of three replicates ± the standard deviation. Statistical analysis was conducted using the One-way ANOVA and post-hoc test (Tukey’s Test). **** denotes *p* < 0.001.

The findings from the optimization of aptamer concentration for the aptasensor using square wave voltammetry (SWV) reveal significant insights into the performance of the sensor. Three concentrations were tested: 0.5 µM, 1 µM, and 2 µM, with mean current of 153.2 µA, 132.1 µA, and 127.9 µA, respectively. A significant current drop was observed between the 0.5 µM and 1 µM concentrations, indicating an enhanced aptamer binding and sensor performance at 1 µM (Fig. 5). However, further increasing the concentration to 2 µM did not result in a significant additional current drop, suggesting that the aptamer binding and sensor performance plateau at 1 µM. Thus, 1 µM was determined to be the optimal concentration for the aptasensor under the tested conditions. These findings are consistent with the principles of aptamer-based sensors, where an optimal concentration is crucial for maximizing binding interactions without causing steric hindrance or saturation effects. The findings align with previous studies that have indicated the importance of optimizing aptamer concentrations to enhance electrochemical responses in biosensors^43^.

### Specificity and selectivity of the aptasensor in bacterial and clinical samples

**Fig. 6:**
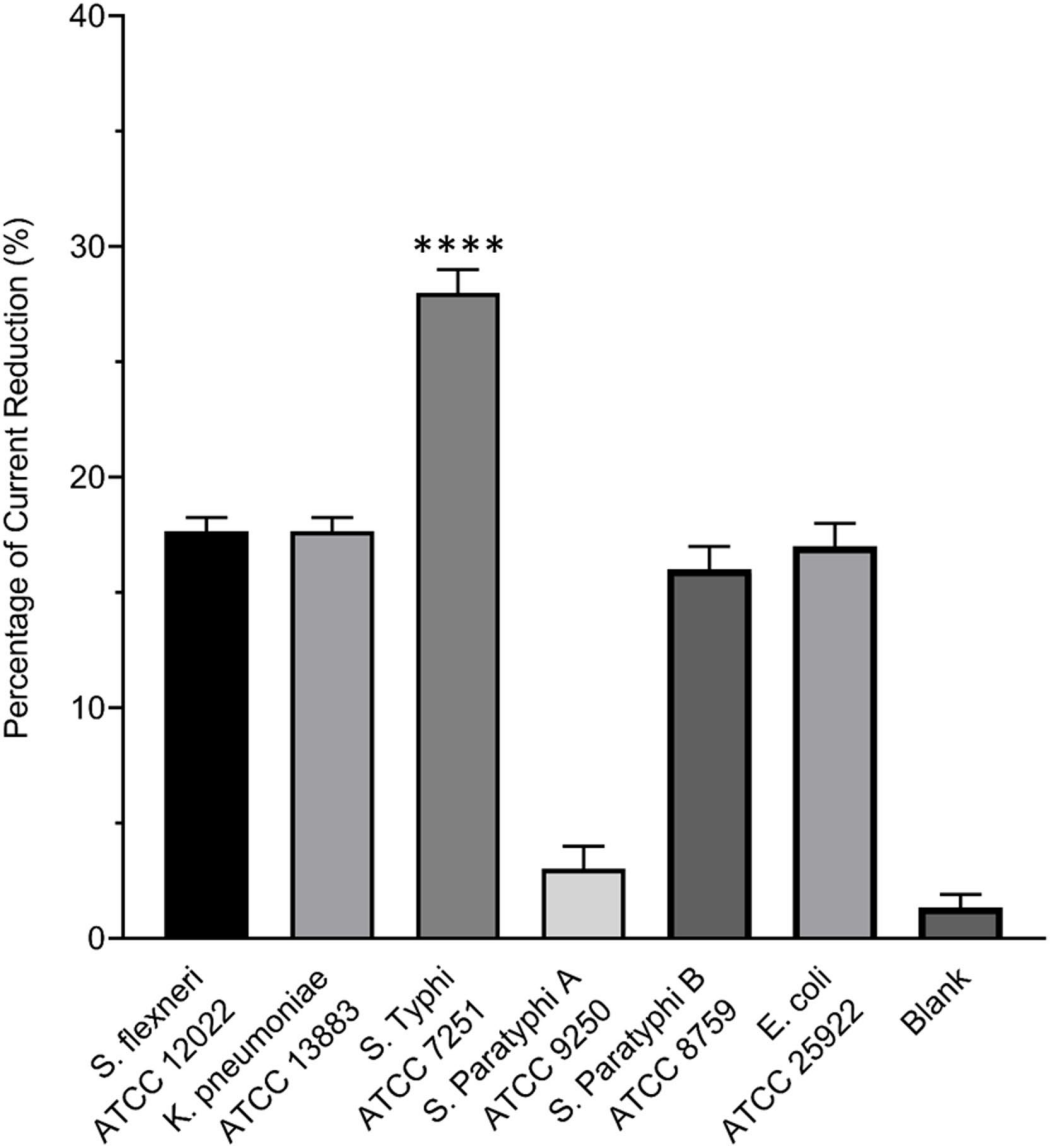
Evaluation of the aptasensor’s selectivity towards *S*. Typhi in comparison to other *Enterobacteriaceae*. All lysates were standardized to the same total protein concentration prior to analysis to ensure signal differences were due to specific binding interactions. Statistical analysis was conducted using the One-way ANOVA and post-hoc test (Tukey’s Test). **** denotes *p* < 0.001.

To evaluate the selectivity of the aptasensor, the current response to the total antigen of several bacterial lysates, including *S*. Typhi, *S*. Paratyphi A, *S*. Paratyphi B, *E. coli*,* K. pneumoniae* and *S. flexneri* was determined using SWV. The mean percentage current reduces 28% for *S*. Typhi, 3% for *S*. Paratyphi A, 16% for *S*. Paratyphi B and 17% for *E. coli*,* K. pneumoniae*, and *S. flexneri* (Fig. 6), demonstrating the aptasensor’s selectivity and its effectiveness in distinguishing between different bacterial lysates. The significantly higher current drop observed for *S*. Typhi compared to the other tested bacteria (>10% difference) indicates that the aptasensor exhibits a strong specificity and selectivity for this specific antigen which is important for applications in clinical diagnostics to avoid false positive results^44^. In addition, the finding of this study in agreement with existing literature that emphasizes the role of aptamers in providing specificity in biosensing applications^45^. For instance, similar studies have reported that aptasensors can effectively differentiate between closely related bacterial species due to the unique binding properties of aptamers tailored for specific targets^46^.

Next, we evaluated the aptasensor’s specificity and selectivity using serum samples from seven typhoid patients confirmed by blood culture method, a healthy individual confirmed negative by blood culture method and six patients with other diseases confirmed by culture methods as reference tests. This will be an important step in verifying the applicability of our aptasensor for clinical use and is an important step in demonstrating feasibility of such device in routing clinical settings. The evaluation showed a sensitivity of 100% in detecting *S*. Typhi in verified blood samples and a specificity of 85.7% (Table 2). The Receiver Operating Characteristic (ROC) curve analysis showed peak current of SWV response lower than 82.8 µA as the best cut-off value to serve as a positive indicator (Figure S4). The Area Under the Curve (AUC) of 0.959 suggests that the aptasensor has a high ability to correctly identify typhoid patients compared to healthy individuals and those with other diseases. This interpretation aligns with established guidelines on ROC analysis, which emphasize that AUC values above 0.80 are generally considered clinically useful, while values above 0.90 indicate very good diagnostic performance^21^. These findings demonstrate the potential of the aptasensor for rapid and accurate detection of typhoid fever, with high sensitivity and good specificity compared to the gold standard blood culture method^47^.

**Table 2:** Diagnostic sensitivity and specificity of the electrochemical aptasensor.

	Blood Culture (+)	Blood Culture (-)	Total
Aptasensor (+)	7	1	8
Aptasensor (-)	0	6	6
Total	7	7	
Sensitivity	100%		
Specificity	85.7%		

### Analytical performance of the aptasensor

**Fig. 7:**
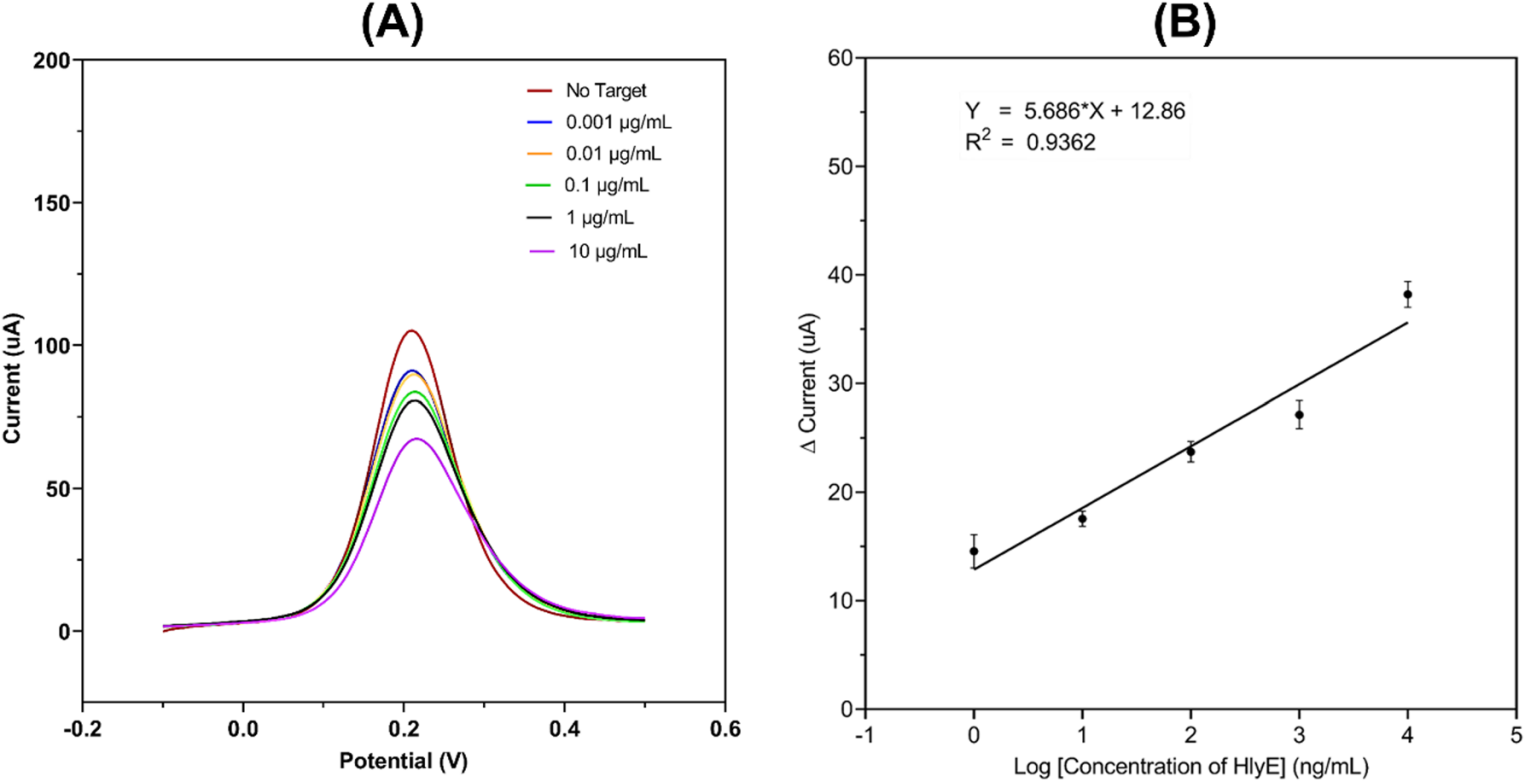
(**A**) SWV response of the developed aptasensor after incubation with different concentrations of target HlyE antigen ranged from 0.001 µg/mL to 10 µg/mL. (**B**) Calibration plot of the aptasensor response to the target HlyE antigen. Error bars represent the standard deviation of triplicate measurements.

Under optimized experimental conditions, the sensitivity of the developed aptasensor was evaluated at various concentrations of the target HlyE antigen ranging from 0.001 µg/mL to 10 µg/mL using SWV measurement. As illustrated in Fig. 7, the peak current of SWV response decreases as the HlyE antigen concentration increases. This result demonstrates a concentration-dependent response behaviour. To determine the LoD and LoQ of the aptasensor, a calibration plot was constructed. The calibration plot shows a linear regression equation of Y = 5.686X + 12.86 with R^2^ = 0.9362. The LoD of the developed aptasensor was calculated to be 0.158 ng/mL. Meanwhile, the LoQ of the developed aptasensor was calculated to be 0.475 ng/mL.

Despite the promising results shown by this newly developed aptasensor, there are several limitations. First, the aptasensor was not fully optimized, which may affect its overall performance. Second, the aptasensor was only evaluated using a small sample size, as this is a feasibility study at the Technology Readiness Level (TRL) 3. Future studies should prioritize optimizing the aptasensor’s test conditions, including the serum dilution factor and target incubation time, to enhance the diagnostic performance of the aptamer. Research has shown that adjusting these parameters can significantly impact the sensitivity and specificity of aptasensors. For instance, optimizing the serum dilution factor can help reduce matrix effects that may interfere with the detection of target analytes, thereby improving the reliability of results^48^. Similarly, the incubation time is critical as longer incubation periods may allow for more complete binding of the aptamer to the target, which can enhance signal intensity and overall assay performance^49^. Moreover, large-scale evaluations of the aptasensor’s diagnostic performance are essential to provide more comprehensive and accurate data. Studies indicate that larger sample sizes improve the statistical power of diagnostic tests, allowing for better assessment of sensitivity and specificity across diverse populations^50^. This approach not only validates the findings from initial studies but also helps in identifying potential variations in performance due to demographic or clinical factors.

While the present study demonstrates high selectivity of the aptasensor toward *S*. Typhi using whole bacterial lysates from multiple clinically relevant species, we acknowledge that specificity was not evaluated against individual purified *S*. Typhi proteins. The use of whole-cell lysates reflects a biologically relevant matrix and provides an initial assessment of selectivity at the organism level; however, protein-level cross-reactivity cannot be fully excluded. Future studies should also therefore focus on evaluating the aptasensor response against purified *S*. Typhi antigens, including lipopolysaccharide (LPS), flagellin (FliC), and other cytolysins, to further confirm molecular specificity and strengthen diagnostic confidence. Collectively, these efforts will contribute to the development of a robust and reliable diagnostic tool for typhoid fever detection.

## Conclusion

The present study demonstrates the development and evaluation of an aptasensor for the rapid and specific detection of *S*. Typhi, the causative agent of typhoid fever. The optimization of aptamer concentration revealed that 1 µM was the optimal concentration. The aptasensor exhibited excellent selectivity, with a significantly higher current drop for *S*. Typhi compared to other bacterial lysates, highlighting its ability to distinguish between closely related bacteria. The lysate-based experiments serve as performance validation under biologically relevant conditions, complementing the purified antigen-based evaluation. The feasibility study using serum samples from typhoid patients, healthy individuals, and patients with other diseases provides a preliminary data on the aptasensor’s potential as a diagnostic tool. The aptasensor achieved a sensitivity of 100% and a specificity of 85.7% compared to the gold standard blood culture method. Furthermore, the developed aptasensor demonstrated a limit of detection (LoD) of 0.158 ng/mL, indicating high sensitivity for target detection. These findings demonstrate the aptasensor’s ability to rapidly and accurately detect *S*. Typhi in clinical samples, offering a promising alternative to traditional diagnostic methods. This aptasensor represents a significant advancement in typhoid fever diagnostics, with the potential to enhance early detection, improve patient outcomes and contribute to public health efforts in combating this disease. Further research and development are warranted to translate this technology into a clinically viable diagnostic tool.

## Supplementary Information

Below is the link to the electronic supplementary material.

Supplementary Material 1

## Data Availability

All data generated or analysed during this study are included in this published article and its supplementary information files.
