# Supplementary material for: Structure-guided development of an electrochemical aptasensor for Salmonella Typhi HlyE antigen detection using in silico and experimental approaches
Source: Sci Rep. 2026 Feb 26;16:11128. doi: 10.1038/s41598-026-38666-6 (PMC13046781; doi:10.1038/s41598-026-38666-6)
Supplement: Supplementary file 1 — Supplementary Material 1 [file 41598_2026_38666_MOESM1_ESM.pdf]

Supplementary Figures

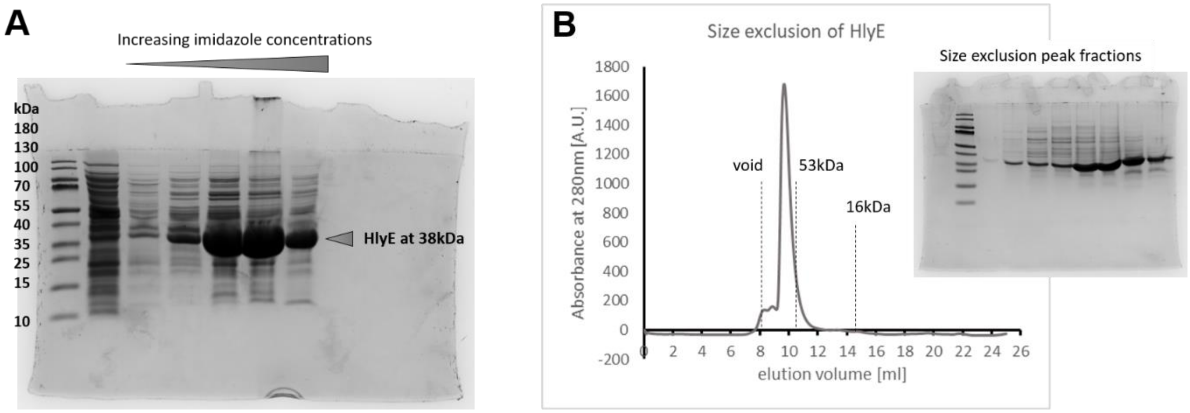

Figure S1: Purification of HlyE protein using (A) IMAC and (B) Size exclusion chromatography. The apparent molecular weight of HlyE in size exclusion is higher than anticipated and suggests oligomer formation.

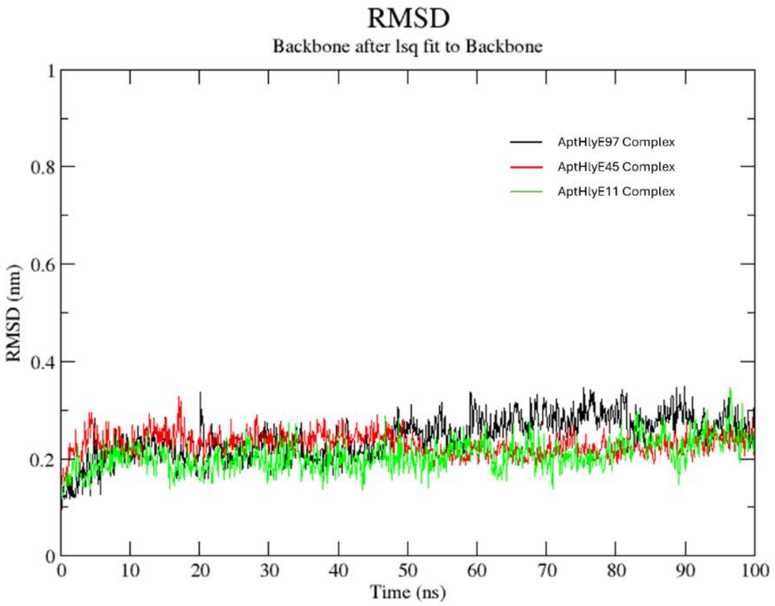

Figure S2: Root Mean Square Deviation (RMSD) plot illustrating the stability of three aptamer-antigen complexes (Complex 11, Complex 45, and Complex 97) throughout 100 ns molecular dynamics simulation.

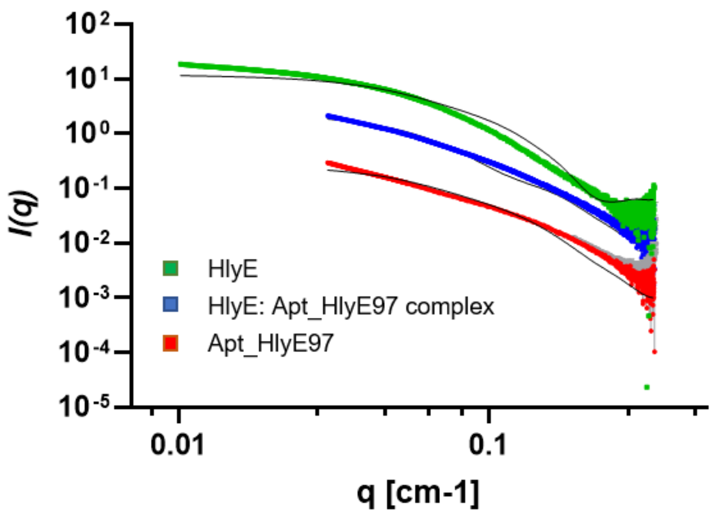

Figure S3: SAXS experimental profiles and theoretical profiles calculated from 3D models of HlyE (green), HlyE:Apt_HlyE complex (blue) and Apt_HlyE97(red).

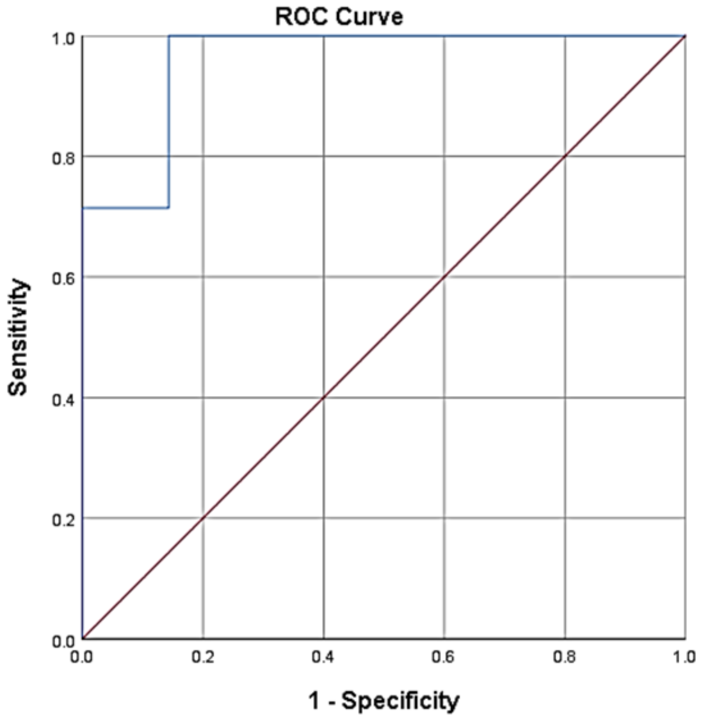

Figure S4: Receiver Operating Characteristic (ROC) curve of the developed aptasensor for typhoid diagnosis. The curve illustrates the trade-off between sensitivity and specificity across various threshold settings. The area under the curve (AUC) indicates the diagnostic performance of the sensor, with an AUC of 0.959, suggesting very good diagnostic performance.

Supplementary Tables

Table S1: The aptamer sequence and primers used for aptamer generation

Aptamer /Primer	Sequences
AptHly E97	5'-$(CH_2)_6$-SH- GGCGAATTCTGGGGCGATATATCCTAGCCGGGAGTCAGTTTAGGACG GGGGCGCGTCCGGGAGGCCAAATAGCCGAATTCGCACG
AptHly E45	GGCGAATTCTGGGGCGATATATCCGGGTTGTTCACTCAGTCTTTGGG GAAGGGTGAGGCGGTCGCCAAATAGCCGAATTCGCACG
AptHly E11	GGCGAATTCTGGGGCGATATATCCTTGATTTTGGCACCGCGGGGTTG TTTAGGGCTATTTGGCTCCAAATAGCCGAATTCGCACG
Forward primer	5’-GGC GAA TTC TGG GGC GAT ATA TCC-3’
Phosphorylated reverse primer	5’PHO-CGT GCG AAT TCG GCT ATT TGG-3’

Table S2: Comparison of experimental SAXS profiles with theoretical SAXS profiles obtained using PEPSI.

Model	Difference Theoretical to Experimental, χ^2 value	Rg [Å] Experimental	Rg [Å] Theoretical
HlyE	114	38.7	29
Apt_HlyE97	22	26	28
HlyE:Apt_HlyE Complex	23	26	29
